# Mr. Shaw's Manual for the Students of Anatomy

**Published:** 1821-12-01

**Authors:** 


*A Manual for the Student of Anatomy; containing Rules for displaying the Structure of the Body, so as to exhibit the Elementary Views of Anatomy, and their Application to Pathology and Surgery.* By John Shaw; being an Outline of the Demonstrations delivered by him to the Students in the School of Great Windmill Street. One closely printed volume, 8vo, pp. 342, 2 plates, London, 1821.

Works of this description do not usually afford materials for analysis, or even criticism. The origin of a muscle, the distribution of an artery, or the processes of a bone, are interesting subjects to the student, who has yet to pass those awful barriers and unknown trials, in Lincoln's-Inn-Fields or Creed Lane; but they have lost their attractions for the veteran Esculapius, whose objects of pursuit are of a different character. The author of the work before us has deviated from the common road, and endeavoured to render his manual more interesting than manuals generally are, by the frequent introduction of physiological, pathological, and therapeutical observations. The utility of this plan, we know, has been called in question—and here, as elsewhere, "much may be said on both sides." For our own parts, we have always been in favour of this mixture of the "utile et dulce," in spite of all the denunciations against it by those stiff-rumped and sour-crout philosophers, whose discourses are as dry as their skeletons—the one being as barren as the other is bare.

We will not assert that the present form and arrangement of Mr. Shaw's book are the very best that could possibly be devised for the London dissector—especially for the student who has but one short year to swallow (for digestion is out of the question) the whole circle of medical and chirurgical science. But we think it a very useful and agreeable pocket companion for the youth who goes leisurely and fundamentally to work, in acquiring a thorough knowledge of anatomy and surgery. We conceive also, that it will prove a very acceptable treat to the provincial practitioner, as a guide in his *post mortem* researches, considerably more adapted to his taste and habits than any thing of the kind that we have yet seen.

One of Mr. Shaw's main objects, in this publication, appears to be the direction of the student's attention *particularly* to those points of anatomy which are most useful, and the recollection of which will be of most importance, when afterwards engaged in the practice of his profession. "In this attempt," says Mr. Shaw, "the student will discover many observations of Mr. Charles Bell;" for which, by the bye, Mr. S. had no great reason to make apology, since the discovery will not be very disagreeable to those who are acquainted (and who are not?) with Mr. Bell's abilities.

Mr. Shaw wisely cautions, though somewhat in jest, the young student, whose time is limited, "not to harrass himself in acquiring such a knowledge of anatomy, as consists in a particular description of the seven and twenty processes of the sphenoid bone, or the exact origins and insertions of the multifidus spinæ," though he disparages not the knowledge of minute anatomy—on the contrary, he contends that no man can be a thorough good surgeon without that knowledge. There is, of course, a wide difference between that minute anatomy which is useful, and that which is devoid of any possible utility.

Some students in anatomy are advised not to read, and this upon the authority of John Hunter. We believe it is the same with the non-reading student, as with the non-reading practitioner, "he blunders through life by rote; or, if he possess native talent, he is often making *fancied* discoveries, which a little reading would have shewn him to be the discoveries of his predecessors."

Mr. Shaw throws out some useful hints to students in his introduction. He is right, we think, in attributing the bad effects of cuts during dissection to bad habit of the living, rather than any thing particularly noxious in the dead body. The best treatment, in his experience, for the inflamed lymphatics and swelled arm, when they do occur, "is to apply lint, soaked in the sugar of lead lotion and tincture of opium, to the arm; and to take calomel purges, and large doses of opium, with plenty of wine and porter." In Paris, we believe, the dissectors there apply the strong muriatic acid to the recent injury, and we believe that inflammation seldom succeeds.

It cannot be expected either by the author or our readers, that we should give any account of the work, as far as relates to practical anatomy. The directions appear to us (who do not pretend to be any adepts in the *art* of dissection, or in the knowledge of *minute* anatomy) to be judicious, and indicative of a thorough acquaintance with the subject. We think it probable that some criticisms will be made on certain descriptions (particularly of the brain) by his brother *artists* of the anatomical profession; but this he much expect. Mr. Shaw has a spice of the critic in his own composition, and consequently he should take in good part the criticisms of others.

Our notices of the work before us must, therefore, be confined to those points of physiology, pathology, and practice, which may prove interesting to the general mass of our readers; and we are not without the hope of being able to offer them food for reflection, as well as matter for practical application, in the course of this analysis.

1. *Hernia.* Mr. Shaw gives many judicious instructions to the young pupil, relative to the dissection of the parts concerned in hernia; and to the practitioner, the following extract may not prove uninteresting.

"The most common seat of stricture in inguinal hernia is the external ring; for though we do not see the ring until we have dissected the parts, still we can feel it, even before the skin is removed, by pushing the finger up along the cord. If the sac has been opened, if the external ring has been cut, and the stricture still continues, what is the cause of stricture? It cannot be produced by the margins of the internal oblique or transversalis muscles, for they will relax. Since we are told by high authority, that the stricture, in such a case, is caused by the internal ring, we cannot deny that it may occasionally happen; but we should be more inclined to say, that the stricture is not caused by the internal ring itself, but by the neck of the sac, which is situated in that part. Our reasons for supposing so, are the following: In the dissection of the parts, in their natural or ruptured state, there is no internal ring, until it is made by pushing up the cellular membrane which surrounds the cord; and even then, if we try its strength, we find it very weak, and particularly on the inner part; while the neck of the sac is generally so strong, that we might as easily break a circle of whip cord as tear it. The external ring, and the neck of the sac, may be considered as the most common seats of stricture; but there are varieties, into the consideration of which it would be impossible to enter at present.' P. 16.

2. *Morbid Anatomy of the Viscera.* A person not much in the habit of opening bodies, is very liable to make mistakes respecting the effects of diseases left after death.

"It is a very common mistake to describe the loaded state of the vessels as an appearance denoting previous inflammation: the state of the true inflamed intestine is so distinct, that it can hardly be forgotten after it has been once seen. In the first stage, there are numerous small vessels seen upon the gut, like those on the eye in ophthalmia, with a suffusion around them; in the second stage, there is matter, or lymph, effused; and in the more advanced state, adhesions are formed between the surfaces of the intestines. But there are many different kinds of peritonitis. In that which is called idiopathic, the peritoneum will be found coated with lymph; but after inflammation, in consequence of strangulated hernia, the substance of the intestine will appear more affected than the proper peritoneum." P. 50.

"From the variety of appearances of inflammation,—from the black spots,—and from the ulceration and corrosion, which, in the course of my dissections, I have seen in the stomachs of those who have died without any marked symptoms of affection of that viscus,—and from the close resemblance which many of these have had to the stomachs of those persons who have swallowed poison,—and from the similarity of the appearances produced by gastritis, and other diseases, to those caused by poison,—I have come to the conviction, that the appearance of the stomach or intestines alone, in a question of poison, is not to be depended on. In the last book which has been written on poisons, (that of Orfila,) the list of appearances which is given, as to be expected, where poison has been taken, corresponds exactly with those which I have found in stomachs where I was certain no deleterious matter had been taken. I am happy to think, that this degree of uncertainty will prevent the anatomist from being called on to decide a question which may involve the life of a fellow creature." 51.

Mr. Shaw properly observes that, in the bodies of children who have died with much intestinal irritation, it is not unusual to find intus-susceptions; but these are seldom the cause of death; whereas, in adults there is generally an accompanying inflammation, producing strangulation, as in those who have died with symptoms of hernia, but with no external tumour. Here we generally find a portion of intestine strangulated by a noose formed of condensed omentum or mesentery—the portion of gut above the stricture being red, thickened, and distended, while the portion below will be pale and empty. Chronic inflammation of the abdomen produces, of course, agglutination of the intestines —"a common appearance in the abdomen of those who have been repeatedly tapped."

"In the greater number of those who die of fever, the intestines appear gorged with blood—not inflamed; but on opening the lower part of the small intestines, we shall generally discover small ulcers, with thickened edges: this appearance is almost always found in the great intestines of those who have died of dysentery." 52.

The most common diseased appearance in the liver is tubercle, which occasionally is found suppurated, the matter penetrating into the contiguous portion of colon. "The pancreas is naturally very firm—whence it is not unfrequently described, by those not familiar with anatomy, as scirrhous, but I suspect that, like the other salivary glands, it is very seldom diseased."

3. *Muscular Fibres in the Urethra.* Mr. Shaw jocosely enough smiles at the discovery of Sir Everard Home, who, by the aid of very powerful microscopes, has recognized MUSCLES in the urethra, whose *tendons* are of the consistence of *mucus.* Mr. Shaw can hardly believe that the spasmodic action of *such* muscles well account for the occasional difficulty of introducing the bougie.

We beg the attention of *all* our young, and a few of our elder brethren, to the following passage—of the truth of which we are convinced, from personal observation.

"It will now be evident that there are several causes of difficulty to the introduction of an instrument through this part of the urethra —1st. The natural curve of the canal—2d. The sinus of the bulb—3. The edge of the triangular ligament: but the principal difficulty is caused by the circular ligament which surrounds the narrow part of the canal.

"It requires so much management, and such a knowledge of the structure of this part, to pass an instrument nicely through it, that I can now, with confidence, assert, that nine cases out of ten of the strictures that are said to exist here, are a consequence of this natural narrowing of the canal having been mistaken for stricture. I am now, by experience, so satisfied of this, that when a patient comes to me complaining of stricture *only at this part,*—if he has been examined by another surgeon, a short time before, I beg him to let the urethra have some days rest before I sound him; for this part of the canal is so irritable, that if there has been the slightest injury done to the membrane, there will be a spasmodic affection produced the moment the bougie touches it, so as to lead the patient to believe that the difficulty of introducing the instrument is in consequence of a stricture. But there is another source of error here,—for the end of the bougie may be indented by being pressed against the edge of the ligament, so as to give exactly that appearance which has been considered as an unequivocal proof of the existence of stricture." 64.

Mr. Shaw observes, a few pages farther on, that although stricture may form at any part of the urethra anterior to the circular ligament, yet it occurs generally at two points:—at an inch and a half from the glans penis, and at six or seven inches down, *i. e.* near the bulb. There are two circumstances not much noticed, Mr. Shaw thinks, which should be borne in mind by the student.

"1st. That there is not one example in a hundred of stricture occurring farther back, than immediately behind the ligament of the bulb.

"2d. That the ducts of the prostate, which are naturally very small, are always more or less enlarged in cases of severe stricture.

"It must be evident that certain practical rules are to be deduced from these facts. 1st. If an instrument is obstructed posterior to the ligament of the bulb, that we may suspect that the cause of the obstruction is not such as will be overcome by the same means as a stricture would; and 2d, We can now understand why, in a severe case of stricture, we ought to be content with so dilating the stricture, as to enable the patient to pass his urine freely,—and that we should not be too anxious to pass an instrument into the bladder, for, in the attempt, the point may enter into one of the enlarged ducts of the prostate, and consequently produce great irritation, and even lead us to suspect that there is still another stricture: if, with this idea, we persevere in pushing the instrument on, we shall certainly do irreparable mischief to the patient." 72.

4. *Iliac Artery.* In the dissection of the thigh Mr. Shaw introduces a long extract from Mr. Charles Bell's "Illustrustrations of the great Operations," lately published, relative to tying the external iliac. As this is an operation now pretty often performed, and as the expense of Mr. Bell's work somewhat limits the range of its circulation, especially through the army, navy, and colonies, where this Journal travels extensively, we shall furnish our readers with several of the rules and directions laid down by the abovementioned able surgeon. Mr. Bell preliminarily remarks that the object of this operation is to tie the artery so high, that the wound shall not interfere with the tumour of the aneurism, nor open the coagulated blood to the influence of the air, nor excite inflammation in the sac by its contiguity. At the same time, there must be no breach of the investing membrane of the abdomen, else the patient's danger will be increased a hundred fold.

"*Incision.* Having ascertained the middle point betwixt the superior spinous process of the os ilii and the symphisis pubis, you feel there the pulsation of the artery. Next feel the spermatic cord, and trace it backwards into the abdominal ring; and mark where it disappears. You have now got two points to direct your incision; make another, by drawing a line from the superior spinous process of the os ilii to the umbilicus; mark a point upon this line, two fingers' breadth from the process. Begin the incision opposite the outer margin of the abdominal ring; carry it over the point where you felt the artery beating, in a direction outward and upward, and let it terminate at the point you have marked at two fingers' breadth from the spinous process of the os ilii, measured in a direction towards the umbilicus.

"'*Second Incision.* Having exposed the aponeurosis, or tendon of the external oblique muscle, and observed the direction of its fibres, pass the directory into the ring, and into the spermatic passage; taking care that the instrument is directly close under the tendon, and, consequently, external to the cord: slit up the tendon in the direction of its fibres.

" '*The Cord.* The spermatic cord is now exposed. With the blunt hook, and the handle of the knife, the cord is to be raised and pressed upward and inward. In doing this, you will necessarily raise the lower edge of the obliquus internus muscle. If the patient be fat, or the aneurism prominent and high, the wound, in this state, will be too confined; and it will be necessary first to pass the directory, and then the point of the finger, under the edge of the muscles, and to divide them in a direction upwards. The condensed cellular membrane, or fascia, which is on the lower surface of the transversalis, will generally yield to the finger.

" 'There will be found a soft mass, just within the Poupart ligament; it may be mistaken for a vessel; the more especially, as the pulsation may be felt on pressing it. It is a lymphatic gland. This gland is to be left in its place. Above this, there is a soft, fatty substance, which is to be put aside with the finger and the handle of the knife; and now, upon putting in the finger, the artery will be distinctly felt.

" 'The space, where you feel the artery, is thus defined: 1. Below, towards the thigh, there is the Poupart ligament, and the internal inguinal gland. 2. On the inside, towards the pubes, you have the epigastric artery. 3. Above, and towards the ilium, there is the edge of the oblique and transversalis muscle. 4. And above, and towards the rectus, you have the spermatic cord.

" 'You should now push up the spermatic cord and cellular membrane,* and you place an assistant's finger there, to guard the peritoneum; you have the epigastric artery on the inside, still involved in its cellular membrane: you may now expose the artery.

" 'Feeling the artery full, and pulsating under your finger, you think it bare; when a little consideration should remind you that it is not.† It is still covered with the sheath, and filaments of the fascia strengthen that sheath: and here I must again observe, that

---

" * To me, it appears that there are good reasons for pushing up the spermatic cord. First, you get much easier at the artery. Secondly, you have the spermatic cord betwixt you and the peritoneum. Thirdly, if you incline, you may, in this direction, push the peritoneum very high, and expose the external iliac artery at its highest point; whereas, if you go above the spermatic cord, and keep it in its place, you must be entangled in the reflection of the vas deferens, and you will make the peritoneum thin as a cobweb, by separating the cellular tissue of the cord from it.

† Mr. Abernethy says, "the pulsation of the artery made it clearly distinguishable from the contiguous parts, but I could not get my finger round it with the facility which I expected." "After ineffectual trials to pass my finger beneath the artery, I was obliged to make a slight incision on either side of it, in the same manner as is necessary when it is taken up in the thigh, where the fascia which binds it down in its situation is strong." This double incision is not necessary in either of these cases; and, I apprehend, very dangerous in the present instance.

the safest way is, to scratch the sheath, directly over the centre of the artery; to cut at the side of the artery is dangerous. The vein lies close by the inside of the artery, and, in some measure, below it. The vein is on the inside, the anterior crural nerve on the outside.* Therefore, I advise you to scratch, until you can pass your probe, or blunt hook, through the sheath and ligamentous fibres which directly cover the artery.

" 'When you have exposed the proper coat of the artery, make the assistant raise the thigh as much as the circumstances of the tumour will admit; then you will be able to grasp the artery betwixt the thumb and the fore-finger; you will find it so loose, that you will experience no difficulty in passing the needle under it. It is struggling to thrust the blunt needle through the sheath and fibres of the fascia, and neglecting to raise the limb, that makes this part of the operation tedious.

" 'One firm ligature of four threads, waxed and oiled, will be sufficient; it is not necessary to tie the artery twice, nor, consequently, to cut it across."† 130.

Mr. Charles Bell long ago observed, that this operation might be expected to be successful when performed for spontaneous aneurism; but not so for aneurism consequent on a wound of the vessel, because in the latter case, the artery must be tied both above and below the wound. The surgical world are aware that Dr. Stevens took up the internal, or posterior iliac artery with success, for an aneurism of the gluteal artery in the case of a negro woman in the West Indies. We do not think that such an operation will ever succeed on the European. We cannot resist the temptation of quoting Mr. Shaw's directions for taking up the femoral artery in the thigh, as performed for popliteal aneurism.

" The limb should be laid rather on the side; a point is then to be marked on the groin, equidistant from the symphysis of the pubes and the superior spinous process of the ilium. Here the artery will be felt. A cord may be fixed at that point, and stretched to the patella; an assistant should then stretch another cord between the superior spinous process of the ilium and the inner condyle of the femur. The centre of the incision should be about an inch above the point where these lines cross; it should be made about three inches long,—not in the line of the fibres of the sartorius, but rather across them. The skin is to be divided in the first incision; and in

---

" * The external iliac vein is close to the inside of the artery. The anterior crural nerve is quite removed from the artery.

† " Mr. John Bell and Mr. Abernethy, and Mr. Maunoir of Geneva, have been advocates for tying the artery twice, and cutting it betwixt the ligatures. It is a practice which may have advantages; but the idea that they thereby made the artery as secure as when tied in amputation, was undoubtedly a great mistake."

the second, the thin superficial fascia, which should be cut to the full extent of the incision through the skin. As the cut is made in a line across the sartorius, there will be little difficulty in recognizing this muscle. (And here I may remark, that none except those that have witnessed the exhibition, can imagine the difficulties which have ensued, in consequence of the edge of the triceps having been mistaken for the sartorius.) The lower edge of the sartorius is now to be raised,—this will expose the fascia which passes from the triceps to the vastus internus; a little perforation is then to be made into the fascia, and a directory is to be passed under it, so that it may be slit up. The sheath which surrounds the artery, vein, and nerve, will now be seen, and when this is opened, it will be easy to pass a blunt needle under the vessels." 133.

The saphena nerve is far enough removed to be in no danger, unless there is great negligence. If tied, it will be a reproach to the surgeon as long as he lives; for the pain will be so distinctly in the line of the nerve as to leave no doubt on the patient's mind as to whom he is indebted for this legacy.

5. *Brain.* Mr. Shaw's chapter on the dissection of the brain will be found very interesting to more than the student—particularly his remarks on the pathological appearances in that important organ. We can glance at but a few of them.

He observes that the appearances said to denote phlogosis of the brain are very questionable, as some of them may be easily washed off. After phrenitis indeed, and violent external injuries, the inflammation will be unequivocal, as the vessels on the surface of the dura mater will be as much blood-shot as the vessels of the conjunctiva in ophthalmia, with layers of lymph occasionally on the inner surface. The other membranes will also be inflamed. Deposits of bone are not uncommonly found in the dura mater, especially in the falx. In three cases, where these were found in contact with the olfactory nerves, the patients had been, for a considerable time previous to death, rendered uncomfortable by the sensation of unpleasant odours. The appearances in apoplexy need not be described here. But Mr. Shaw remarks on the importance of observing how the blood is diffused over the brain, in injuries, "as it will shew the inutility of puncturing the dura mater after the trepan, with the intention of evacuating blood which may be under it."

The tunica arachnoides will be found thickened in all cases where inflammation of the brain has existed for some time, and there will also generally be effusion of serum under the membrane.

"It is, perhaps, improper to attach much importance to this effusion, because it is found in almost every case of protracted disease,—as in fever, or in cases where a patient has died in consequence of irritation of any viscus, and particularly after any operation on the bladder, or from retention of urine. When we find this effusion, we may predict that there will be water in the ventricles." 159.

The gorged state of the vessels of the pia mater is often owing to the position of the head after death; "but in the true inflammation, the vessels of the pia mater will be very numerous, and the membrane will be found thickened."

The substance of the brain, in the infant, is very soft, and gradually becomes firmer until extreme old age, when it becomes soft again.

"It is very difficult to determine whether the great fullness of the vessels is to be taken as denoting that there has been any particular action in them during the life of the patient; because there is frequently an unnatural degree of fullness to be found in the vessels of the brain of persons in whom there were no symptoms of deranged functions during life.—I am, therefore, inclined to consider the fullness of the vessels, in the greater number of cases, to be in a great meaure dependent on the position of the head after death, and particularly in those cases of fever, where, in consequence of the blood not coagulating, it flows freely up by the deep veins, in which the valves are generally so imperfect, as to permit the blood to pass. We may often see a proof of this, in the quantity of blood which escapes after the brain is removed, if the head be left in a depending position." 160.

The substance of the brain, Mr. S. has found to be very tough and firm in those who have died maniacal, with the convolutions on the surface peculiarly distinct. "After epilepsy, we may expect to find solid tubercles in the substance; but I have generally found them near the base of the brain." If a person has been blind of one eye, the corresponding optic nerve, which is frequently small and transparent, should be traced to the thalamus, to endeavour to ascertain the decussation—if it do exist, for it is still a question. In Mr. Shaw's experience, when the left eye was affected, the right tractus opticus was smaller and more transparent, and vice versa. Mr. Shaw doubts whether a moderate proportion of water in the ventricles, base of the brain, and theca vertebralis, be certainly a morbid phenomenon. In the prosecution of experiments on the spinal marrow of the ass, he has had occasion to open the sheath several times between the occiput and atlas; and in every instance, immediately on puncturing it, about two ounces of clear limped fluid escaped in a stream. This he has noticed in a proportionate degree in other animals.

Mr. Shaw suspects that the inflammatory appearances described in the spinal brains of tetanic patients have been produced by the gravitation of the blood after death. Mr. S. examined a man lately who sunk under tetanus. He got him placed on his face immediately after death, and on opening the spine there was no appearance of that loaded state of the vessels on the posterior column, which has been considered as a proof of the previous existence of inflammation of the spinal marrow. But the anterior portion, which in this case had been the most depending part while the blood was gravitating, was covered with a congeries of distended vessels. Mr. Shaw, however, does not deny the occasional inflammation of the spinal marrow, or the existence of tumours in it, for he has often seen the latter.

Mr. Shaw throws out several interesting hints, of a forensic nature, relative to examinations of the head in cases of sudden death. He properly observes, that it is sometimes exceedingly difficult to ascertain whether many of the appearances, within the cranium, be attributable to injury, previous disease, or *post mortem* changes. Even the question whether or not there has been a fracture of the skull, before death, is not always so easy to be decided as one would imagine.

"If the fracture has occurred immediately before the patient's death, there will be coagulated blood found upon the bone, and in the fissures; but if the patient has survived for some time, there will be marks of inflammation, and perhaps pus in contact with the scull. If a fracture has been produced in making the examination, (which sometimes happens even in a very careful dissector's hands,) the blood in the fracture will not be coagulated, nor will there be any effusion around the portions. If there have been symptoms of fracture after a blow on the upper part, and if we cannot discover one opposite to the part struck, we should look to the temples, or to the base of the scull." 164.

We are tempted to quote the following forensic case, in which Mr. Charles Bell was concerned, as affording materials for reflection to the medico-legal and pathological inquirer.

"An industrious man returning home from his work, found his house empty of every thing,—the bed he was to lie upon, and the tools of his trade, sold for gin by his wife, whom he found in a gin-shop, where she had been drinking and dancing. He brought her home, and, in the passage of his house, struck her, and ordered her to go up stairs. She refused to go; he carried her upon his shoulders, and the contention continuing up stairs, he struck her again. There having been no one present, we have only the husband's account of her death. He said, that whilst sitting on her chair, she fell down, upon which he threw her on the bed, conceiving that she was in a fit, such as he had seen her in formerly. Some of her neighbours coming in, found her dead. Mr. C. Bell was requested to examine the body of this woman. The man was afterwards tried at the Old Bailey, for murder, and Mr. Bell's deposition was nearly to this effect. In the abdomen and thorax, nothing appeared remarkable, further than that the stomach contained a quantity of gin; and that there was a blush of redness on the lower orifice of the stomach and duodenum. On the head, there were several bruises; but the bone was not at all hurt, and no extravasation appeared under the bone. On exposing the membranes of the brain, the vessels of the pia mater were empty of blood, as if from pressure. There was a serous effusion under the tunica arachnoidea, and in the cavities of the brain, similar to what has been found in those who have died from intoxication. On the surface of the brain, there were what appeared to be spots of extravasated blood; but upon tracing them towards the base, they proved to be streams of blood which had flowed from a vessel ruptured in the base of the brain. The base of the brain was covered with coagulated blood, in which also, all the roots of the nerves were involved. On dissecting the cavities of the brain, the blood was found to have penetrated into the third ventricle, by perforating its floor. Upon taking out the brain, and tracing the vessels in the base, the anterior artery of the cerebrum going off from the internal carotid of the left side, was found torn half way across: from this source came the extravasated blood.'

"The cause of this woman's death, was the bursting of the blood from the ruptured vessel, and the pressure on the brain, or, more correctly speaking, on the vessels of the brain. As to the cause of the rupture, Mr. Bell's opinion coincided with the best authorities in pathology, that there is a state of the vessels, in which an external injury or shock is more apt to produce rupture,—and drunkenness may be supposed to be the artificial state of excitement, which most resembles this state of the vessels. Being asked whether the blows were the cause of the rupture? he said he conceived it very likely that a shock would rupture the vessel: and being then asked, whether he conceived that this woman was more likely to have a vessel ruptured, from having been intoxicated? he was of opinion, that intoxication, and the struggle, were likely to produce such a degree of activity of the circulation in the head, that a less violent blow might produce rupture, than what, in other circumstances, would have proved fatal."

"The man was acquitted." 165.

6. ***Pathological Examination of the Chest.*** The pleura, of course, is the first part that presents for examination. Serous effusion, between the pleura, Mr. Shaw truly remarks, is a very common appearance in the greater number of cases where there has been protracted visceral disease, and in children who have died of measles. Deaths, from cancer, have been sufficiently numerous, in Middlesex Hospital, to prove that serous effusion in the chest is the most common immediate cause of the fatal termination in that terrible disease. It is also not very uncommon after severe accidents or great surgical operations. If a patient die of acute thoracic inflammation, we shall find a quantity of coagulable lymph or inflammatory crust on the inner surface of the chest, of the consistence of jelly. These exudations approach, in advanced stages, to the form of natural membranes, but thickened and opake. Adhesions between the pleura costalis and pulmonalis can hardly be called a disease, but the sequela of inflammation, and of very little consequence. In examining the lungs, the effects of sanguineous *gravitation* should not be mistaken for sanguineous *congestion*, as is sometimes done. In the *former* case there will be found no crowd of fine vessels filled with blood, or any other mark of inflammation of the pleura. The blood accumulated from gravitation is of a dark colour —in inflammation, the part will appear florid.

" The lung which is loaded with blood, from gravitation, may be distinguished from that which is condensed by inflammation, by cutting into it; for then, the blood may be squeezed out, and the lung will regain its natural appearance;—but a diseased portion will feel denser and heavier, and when squeezed, the blood will not escape, nor will there be any of that crackling feel, which is felt in the healthy structure; and the interior of the substance, when it is cut into, will have much resemblance to the liver,—whence it has been called, by the French pathologists, *pulmo hepatizè*." 199.

The appearances in the lungs, where the patient has died of phthisis, are unhappily too familiar to us all. After describing tubercles, induration, vomicæ, and abscesses, Mr. Shaw observes, that " the state of the large vessels, in these great abscesses, is very extraordinary; for they will sometimes be found with open mouths, projecting into the sac—more commonly, however, with their mouths plugged up with coagula, like the arteries of a stump after amputation."

" Tumours will sometimes be found projecting from the surface of the lungs, and widely interspersed in their substance, of quite a different texture from tubercles, being of a very vascular and porous, or cellular nature; perhaps these may be called *sanguineous tumours.* Those upon the surface, are of a reddish colour, and are covered with a smooth membrane. These are often found in those subjects, in which there is a similarly diseased structure in the liver and lymphatic glands, and in the substance of the testicle. Indeed, when the lungs are diseased, we generally find that the lymphatic glands, and particularly the mesenteric glands, are in the same state. There is one species of tubercle that is very rarely seen,—viz. a soft pulpy tubercle, of a light brown colour." 200.

In *broken-winded* horses, and in some asthmatic subjects, the air-cells are occasionally found ruptured, and several of them communicating and forming a large cyst. This was the case in the lungs of our great lexicographer, Dr. S. Johnson, and is particularly described by Laennec. Earthy concretions are very commonly found about the roots of the lungs, especially in scrofulous subjects. Where cancer of the breast has extended to the bone, the lung beneath will generally be found affected also, particularly where the cancer has been of that kind denominated "*fungus hæmatodes.*" If a patient die of irritation in the larynx, the lungs will be found congested, and will not collapse. In croup, the membrane may be traced into the branches of the bronchia, and the cells are filled with purulent matter.

The pericardium, our author observes, is very frequently found adhering to the heart. "If," says he, "we compare the number of cases which are now seen of this (which is the consequence of violent inflammation of the internal surface of the pericardium) with the importance which the older authors attached to the few cases recorded, we should be inclined to say, that the disease of pericarditis must be much more common now than formerly." We think there can be no doubt on the subject. In chronic cases Mr. Shaw has found the coagulable lymph a quarter of an inch in thickness, and in several distinct layers, the most internal of which he has been able to inject. Where patients are cut off in thirty or forty hours, we shall generally find only a very delicate layer of lymph between the heart and its covering.

Mr. Shaw appears to restrict the term *aneurism* of the heart to those very rare cases where there is "a projecting tumour from the side of one of the ventricles." The appellation of aneurism, however, has been so long and so generally applied to enlargements, active and passive, of that organ, that it is hardly safe to displace it all at once. We think, indeed, that the term *hypertrophia* of Laennec, is superior to any other designation. On what have been called polypi of the heart, Mr. Shaw makes some pertinent observations, which we shall present to our readers.

"That these *polypi* are, for the most part, formed after death, there can be little doubt; but still there are circumstances which have induced many to believe, that they are formed during life. They are often found in layers; and this, it is said, argues a successive formation; or they are attached to the sides of the arteries, where their coats are diseased, and their attachment does not appear to be accidental, or owing to the simple coagulation of the blood. In many instances, however, when these coagula are remarkably firm, and such as we might suppose were formed during life, we shall find, upon examination, that the extremity, which is loose, lies in a direction contrary to the course of the blood; a direction, in which we must be sensible it could not have remained during life, for it must have been driven in the direction of the current of the blood, while the root was held nearer the heart. In the centre of many of these coagula, there is an oily fluid, so similar to pus, that I have seen such cases exhibited, as examples of abscess in the interior of the heart." 203.

Of malformations of the heart we shall take no notice. Mr. Shaw thinks that ossification of the aortic valves (one of the most common of the valvular diseases) is so far a natural consequence of old age that it does not produce much distress in a person above the age of sixty; "but in a young person, whose left ventricle is still in a vigorous state, the consequences are terrible; for we find the heart sometimes increased to nearly the size of that of a bullock, and bearing evident marks of inflammation." 204.

Mr. Shaw questions the propriety of ascribing angina pectoris to ossifications of the coronary arteries, since in many old people, who never had the slightest symptom of this disease, he has found the coronary arteries like tubes of bone through their whole course. We believe that the symptoms designated by the term angina pectoris are not now generally attributed exclusively to ossification of the coronary arteries—but to a debility of the central organ of the circulation, by whatever cause induced, which disqualifies it for performing its function, at times, and especially when any exertion is used in going up an ascent. In short, many organic diseases of the heart and its vessels and valves will produce all the phenomena of angina pectoris. Mr. Shaw has found, what corresponds with the best observers, that when the heart is very large, there is generally a stricture of the aorta. "In such a case, the state of the heart is very analogous to that of the bladder when there has been a stricture of the urethra." Those dilatations of the aorta, so common in old people, Mr. Shaw thinks, may be the primary state of an aneurism. "For if we minutely examine an artery which is dilated, we generally find one point thinner than the rest, which, had the patient lived longer, would probably have given way, and then an aneurismal tumour would have been formed at the part."

Mr. Shaw combats the principle of Dr. Jones, that the middle and internal coats of arteries should be cut through by the ligature, to facilitate, or rather secure the obliteration of the arterial tube. Our readers are all aware that a very slight pressure indeed on an artery will quickly arrest the flow of blood through it, as effectually as if tied with the greatest degree of force. Mr. Shaw contends, moreover, that the vessel must be much weakened by the process of Dr. Jones, since blood may escape (as injection does in the dead body) by the side of the ligature into the neighbouring cellular structure.

7. *Nervous System.* In those parts of the work, particularly where the nervous system is the subject, we trace the ideas of Mr. Charles Bell—a circumstance which not a little enhances the value of the publication, in our eyes, and which induces us to go much farther in this analysis than we otherwise would be inclined to go. Another reason why we protract this article is, that the book cannot be expected to travel in the main circuits of this Journal, its destination being principally for the student, as its title imports; consequently its pathological and physiological ingredients will be directly disseminated through the present medium, to an extent which they could not otherwise reach for years—and then through circuitous channels. We are not without hopes, also, that the specimens herewith laid before the public, will tend to increase the circulation of the work itself, among a class of medical society for which it seems little adapted by its title.

Speaking of the par vagum, and its numerous connexions in thorax and abdomen, Mr. Shaw introduces the following note, which we shall quote.

"The par vagum connects the larynx, pharynx, lungs, heart, and stomach; and the sympathies it produces in health and disease, are very many. Disorder of the stomach deranges the secretion of the larynx; a vomit, or nauseating medicine will loosen the viscid secretion of the larynx and pharynx; disorders of the stomach, acting through the pulmonic plexus, will occasion cough; and medicines acting on the stomach, will alleviate asthma. Through the plexus of this nerve, the heart and lungs are united, ever corresponding in action. When life seems extinguished by suffocation, (in experiments on animals) pricking the heart will be followed by respiration; and in the apparently drowned, the play of the lungs, in artificial breathing, brings after it the action of the heart. It is well known how disease of the lungs affects the heart; but it is not so generally observed how much disease of the heart resembles pulmonary disease.

"Looking to the distribution of the par vagum on the stomach, and the plexus of the nerve, in its course upon the œsophagus, it will not appear surprising, that disorder of the uterine system, affecting the stomach, and also primary disorders of the stomach itself, should produce the *globus hystericus*, or paralysis, or spasms of the pharynx and œsophagus. Although the heart and stomach be separated by the diaphragm, yet through this nervous cord they are united: and this explains why disorder of the stomach should produce such changes on the heart's action. The pause, or intermission of the pulse, which, in many diseases, is a fatal symptom, is often produced in a manner less alarming—merely by irritation of the stomach. Seeing these many connexions of the stomach with the vital parts, through this nerve, our surprise ceases at a blow on the stomach proving instantly fatal." 248.

Mr. Bell, in his lectures, has, for some time past, shewn that all the spinal nerves, the sub-occipital, and the *fifth*, have several essential circumstances in common—for instance, they have each two distinct roots, with a ganglion on one of these roots—that they are exquisitely sensible—that they are distributed to muscles for locomotion and action—that each nerve is distributed to its corresponding division of the body, without ever taking a longitudinal course thither.

"When we examine the origin of the nerves minutely, we shall find, that the Vth is the only nerve of the scull, which comes off in such critical circumstances, as to have a root from the crus cerebri, and another from the crus cerebelli,—which parts may, by comparative anatomy, be proved to be the continuation of the anterior and posterior divisions of the spinal marrow. The Vth will also be found to be the only nerve within the scull, which has a ganglion at its roots. Those who have dissected the deep nerves of the head, or who have attempted to demonstrate the branches of the Vth pair to students, will be able to estimate the value of this view.

"I have examined the nerve repeatedly, in its whole course, in man, in the horse, the ass, the calf, and the dog. By these dissections, I have been convinced, that, in every respect, the Vth pair resembles the spinal nerves, even in the peculiar form of its ganglion and plexus. In the horse, there is as distinct a plexus formed by the branches of this nerve which go to the different parts of the head, as there is formed by those which go from the axilla, or loins, to supply the limbs. I conceive, also, that the form of the part from which this nerve arises, is analogous to that of the spinal marrow where the axillary nerves take their origin. If this be correct, it will be another proof of the similarity of the Vth nerve in the spinal nerves." 256.

In this investigation, Mr. Shaw, has had to correct the very common mistake that the great sympathetic has its principal connexion with the nerves of the head, through the medium of the VIth pair. The branches which *appear*

to go to the VIth pair, go, in reality, to the ganglionic portion of the Vth. By the establishment of this fact, Mr. Shaw thinks it is proved that even the connexion between the sympathetic and the Vth is similar to the union of the sympathetic with the ganglionic roots of the spinal nerves.*

Mr. Shaw makes many ingenious remarks on the nerves of the face, especially the portio dura of the auditory nerve and its connexions, but we cannot transcribe or abbreviate them. Upon the great sympathetic and its connexions also, much interesting matter is introduced. We shall find, he observes, that each cervical nerve has a double root—*i. e.* one from the anterior, and the other from the posterior column of the spinal marrow—that the one from the *posterior* has, immediately before it joins with the anterior, a ganglion formed upon it; and if we carefully examine this, we shall find that, from each ganglion, a small nerve is sent off, to unite with the sympathetic. This circumstance was entirely overlooked by Bichat, who has described the ganglion, but not the nerve of communication. Had this great anatomist lived to investigate farther, he would probably have given up the idea of the sympathetic being a part entirely distinct from the system of the spinal nerves.

Mr. Shaw's chapter on the surgical dissection of the neck and head contains matter that every surgeon should carefully study.

And here we must close our notice of the work, having passed over entirely the great body of it, as occupied in anatomy and physiology, on which we could not enter. We shall not go the length of one of our cotemporaries, in asserting that Mr. Shaw's book will supersede all others of the kind, because we are convinced that, in the present state of things, such an event cannot happen, even if the Angel Gabriel were to write a student's manual. On the contrary, we are disposed to think that the work will be more popular among those classes for whom it was least designed—namely, those at a distance from the metropolis, who are slowly initiating themselves, at county hospitals and infirmaries, in the knowledge of anatomy and dissection—or who, having concluded their studies, may be anxious to profit by all the chances of *post mortem* investigations, which private or public practice may throw in their way. We repeat it, as our opinion, that the work is too good for the *migratory annual* student, who thinks his time will not

* See a paper in the Phylosophical Transactions of the present year on the subject.

admit of more knowledge than the most laconic manuals of anatomy can supply. To this class (unfortunately a large one) we fear Mr. Shaw's book will not prove quite satisfactory; but he may well console himself by reflecting and knowing that his labours will circulate widely among those who are most capable of rightly appreciating their value.

———Neque te ut miretur turba labores. *Hor.*

